# Impact of Folate Intake on Bone Mineral Density in Patients with Inflammatory Bowel Disease

**DOI:** 10.3390/nu16010006

**Published:** 2023-12-19

**Authors:** Alicja Ewa Ratajczak-Pawłowska, Aleksandra Szymczak-Tomczak, Michał Michalak, Anna Maria Rychter, Agnieszka Zawada, Kinga Skoracka, Agnieszka Dobrowolska, Iwona Krela-Kaźmierczak

**Affiliations:** 1 Department of Gastroenterology, Dietetics and Internal Diseases, Poznan University of Medical Sciences, 61-701 Poznan, Poland; aleksandra.szymczak@o2.pl (A.S.-T.); a.m.rychter@gmail.com (A.M.R.); aga.zawada@gmail.com (A.Z.); kingskoracka@gmail.com (K.S.); agdob@ump.edu.pl (A.D.); 2 Poland Doctoral School, Poznan University of Medical Sciences, 61-701 Poznan, Poland; 3 Laboratory of Nutrigenetics, Department of Gastroenterology, Dietetics and Internal Diseases, Poznan University of Medical Sciences, 61-701 Poznan, Poland; 4 Department of Computer Science and Statistics, Poznan University of Medical Sciences, 61-701 Poznan, Poland; michal@ump.edu.pl

**Keywords:** folic acid, osteoporosis, inflammatory bowel disease, bone mineral density, Crohn’s disease, ulcerative colitis

## Abstract

Background: Decreased bone mineral density (BMD) is a common problem among patients with inflammatory bowel disease (IBD). We hypothesised that an insufficient intake of folate might affect BMD. Methods: The study subjects included 26 with Crohn’s disease—CD, 30 with ulcerative colitis—UC, and 31 healthy adults (control group—CG) aged 18–50 years. Participants were asked to follow their usual diet, and dietary intake was assessed by a 4-day, 24 h dietary recall. All the participants filled in a questionnaire referring to folic acid supplementation. The BMD, T-score, and Z-score of the lumbar spine (L1–L4) and femoral neck (FN) were assessed. Results: We found significant differences in the body mass, BMI (body mass index), CRP (C-reactive protein), BMD, Z-score, and T-score of the L1–L4 and FN between groups. There were no differences in energy and folate intake or the percentage coverage of recommended dietary allowances (RDA) of folate in all groups. Moreover, 70% of patients with UC, 92% of patients with CD, and 77% of CG patients showed insufficient folate intake. Folic acid was supplemented with a similar frequency in patients covering and not covering the RDA of folate. The intake of folate per 1000 kcal correlated positively with the CD group’s BMD and T-score of L1–L4. Conclusions: Insufficient folate intake is common in patients with IBD and healthy individuals. The impact of folate on BMD in IBD is not clear. We need more studies on the association between folate intake, folic acid concentration, and BMD in IBD.

## 1. Introduction

Folic acid plays many functions in the human body. Firstly, it is responsible for DNA methylation [1]. Secondly, folic acid deficiency leads to megaloblastic anaemia. Moreover, it prevents neural tube defects, and, therefore, folic acid supplementation is recommended during preconception and pregnancy [2,3].

Next, folate participates in homocysteine metabolism and decreases its concentration [1]. The study showed that folic acid supplementation in doses of 0.5–5 mg daily may decrease the homocysteine concentration by about 25% [4]. Folic acid, vitamin B6, and vitamin B12 are well-known factors reducing homocysteine levels. However, it is vital to note that vitamin D may also support vitamin B6, B12, and folic acid action [5]. Homocysteine is a well-known risk factor for cardiovascular disease [1]. However, recent studies have shown that homocysteine may also affect bone tissue [6]. According to Herrmann et al., homocysteine increases osteoclast activity, decreases osteoblast activity, affects the bone matrix, and reduces blood flow in bones [7]. A decrease in the homocysteine level may also result from variants C → T in the *MTHFR* (methylenetetrahydrofolate reductase) gene. The homozygotes *MTHFR* 677TT presented a lower activity of MTHFR, leading to hyperhomocysteinemia. Vitamins B6 and B12 are also involved in homocysteine metabolism, and a deficiency of them may affect the homocysteine level [8].

Moreover, folic acid also affects bone turnover. Salari et al. investigated the impact of 6-month folic acid supplementation on bone turnover. They observed that the serum levels of osteocalcin decreased non-significantly in the study and control groups; however, the differences between the groups were significant after six months of the study [9].

On the other hand, in an observational study, there was no correlation between the folic acid level and bone loss [10]. It is vital to notice that folate is involved in epigenetic changes, which influence interactions between the gut microbiota and the immune system. For these reasons, a low folic acid level may increase the risk of inflammatory bowel disease (IBD) development, including Crohn’s disease (CD) and ulcerative colitis (UC) [11].

On the other hand, a meta-analysis, including twelve studies, indicated that patients with IBD presented lower folic acid levels than healthy adults [11]. This low folic acid concentration is multifactorial and is related to, e.g., malabsorption, higher demand, insufficient intake, or pharmacological treatment [12]. However, data about folate intake among patients with IBD are contradictory. The meta-analysis showed no significant differences in folate intake between the study and control group [13]. The risk factors for folic acid deficiency are especially sulfasalazine and methotrexate treatment and the activity of the disease, affecting malabsorption and insufficient intake [14,15].

Moreover, folic acid has a role in colorectal cancer prevention [16] and for this reason is especially important for patients with UC. According to the European Crohn’s and Colitis Organisation, the toxicity of methotrexate may be decreased by co-prescription folic acid [17]. One of the most common extra-intestinal complications of IBD is low bone mineral density (BMD), leading to osteopenia and osteoporosis. Their pathogenesis is multifactorial, including an insufficient intake of nutrients [18]. The risk factors of osteoporosis in IBD are, among others, chronic inflammation, steroid therapy, malabsorption, low body mass, and diet [19]. Many studies focus on the impact of vitamin D and calcium intake or supplementation on BMD in IBD [20,21,22,23]. However, we need to look for new factors that affect bones to reduce the risk of osteoporosis and improve the care of patients with IBD.

Folate can be found in nuts, eggs, green leafy vegetables, or products enriched with folate (e.g., bread or rice in some countries). The bioavailability of folate in products is lower than in supplements. It is also vital to notice that patients with IBD do not tolerate some products rich in folic acid, and these may be eliminated from their diet [24].

Although some studies refer to folic acid intake in inflammatory bowel disease, no studies have focused on folic acid intake and its impact on bone mineral density in inflammatory bowel disease.

This study aims to look for a relationship between folate intake, folic acid supplementation, and BMD in patients with CD and UC. We hypothesised that an insufficient intake of folic acid might affect BMD.

## 2. Materials and Methods

The study subjects included 56 patients with IBD (26 with Crohn’s disease—CD and 30 with ulcerative colitis—UC) and 31 healthy adults (control group—CG) aged 18–50 years, recruited at the Department of Gastroenterology, Dietetics and Internal Medicine (Poznan University of Medical Sciences, Poznan, Poland) between 2020 and 2021. IBD diagnosis among patients was based on endoscopic, histopathological, and radiological criteria. Additionally, patients were treated according to the standards of the Polish Society of Gastroenterology and the guidelines of the European Crohn’s and Colitis Organisation (ECCO). The exclusion criteria were pregnancy and diseases that may affect the BMD (such as celiac disease, diabetes, kidney disease, and liver diseases) and treatment with methotrexate and sulfasalazine. Participants did not have any specific diet—they were asked to follow their usual diet and not make any changes during the study. All participants provided their written informed consent. The local bioethics committee approved the study (Approval No. 92/09 and Resolution No. 39/20 on 16 January 2020).

Participants were asked to follow their usual diet, and dietary intakes were assessed by a 4-day, 24 h dietary recall. The DIETA 6.0 food analyser analysed data from the dietary recall. And results referring to the intake of folate, energy, and covering the recommended daily allowance of folic acid were obtained. Additionally, we calculated the amount of folate intake per every 1000 kcal consumed. Analyses were carried out for women and men.

Moreover, all participants filled out a questionnaire referring to folic acid supplementation. We collected blood samples for the assessment of CRP (C-reactive protein). 

We also assessed the BMD, T-score, and Z-score of the lumbar spine (L1–L4) and femoral neck (FN) by the DXA (dual-energy X-ray absorptiometry) method using the Lunar DPX-Plus device (Lunar Inc., Madison, WI, USA). Additionally, we assessed body mass and weight, and based on them, we calculated the BMI (body mass index).

### Statistical Analysis

The numerical data did not follow a normal distribution (the Shapiro–Wilk test), so the descriptive statistics are presented as medians and interquartile ranges (Q1–Q3). The categorical data are presented as numbers and relative frequencies n (%). The comparison between the two groups was performed using the Mann–Whitney test. In cases where more than two groups were analysed simultaneously, the Kruskal–Wallis test was used. Furthermore, we used Dunn’s post hoc tests to denote homogenous groups for significant results. The categorical data were analysed by a chi-square test for independence. Spearman’s correlation coefficient assesses the statistical dependence between two variables. The *t*-Student test checks its significance.

The data were analysed using the statistical package TIBCO Software Inc., Palo Alto, CA 94304, USA (2017) on Statistica version 13.3.704.0 (data analysis software system) (http://statistica.io, accessed on 28 June 2017). All tests were considered significant at *p* < 0.05.

## 3. Results

We did not find significant differences in folate (*p* = 0.23) and energy intake (*p* = 0.58) or meet the RDA (*p* = 0.23). It is vital to note that only 57% of patients with CD, 68% of patients with UC, and 77% of healthy adults meet the RDA. The patients with CD and UC presented a lower BMD, T-score, and Z-score of the femoral neck and L1–L4 than the control group. Moreover, the patients suffering from CD and UC had a lower body mass and BMI compared with the control group (Table 1).

There are no significant differences in meeting the RDA for folate and the intake of energy and folate per 1000 kcal between the groups in both women and men. The highest percentage of participants meeting the RDA among women was in the UC group. On the other hand, among men, the highest frequency of meeting the RDA was in the control group. The healthy women consumed more calories than those with CD or UC, but these differences were insignificant. On the other hand, the men with UC consumed more calories than the men with CD and the men in the control group; however, this observation was not statistically significant (Table 2).

Table 3 presents differences in the frequency of folic acid supplementation between groups with an adequate and inadequate intake of folate.

Moreover, we did not find significant differences in the BMI, CRP, BMD, T-score, and Z-score of L1–L4 and the femoral neck between groups with insufficient and normal folate intake in any of the groups (Table 4). 

Additionally, we find no differences in the BMIs, CRPs, BMDs, T-scores, and Z-scores of L1–L4 and the femoral neck between the patients with UC and CD supplemented and not supplemented with folic acid (Table 5). Only one person was supplemented with folic acid in the control group. Therefore, comparing the supplemented and the non-supplemented groups was impossible.

We found a positive correlation between BMI and folate intake (r = 0.52; *p* < 0.01), the % coverage of RDA (r = 0.52; *p* < 0.01), and the intake of folate per 1000 kcal (r = 0.45; *p* = 0.02). Additionally, among the Crohn’s disease group, the folate intake per 1000 kcal correlated positively with the BMD (r = 0.40; *p* = 0.04) and T-score of L1–L4 (r = 0.42; *p* = 0.03).

We did not find a correlation between the bone mineral density, T-score, and Z-score of the lumbar spine (L1–L4) and FN and folate intake.

## 4. Discussion

In our study, we looked for an association between folate intake and BMD in patients with IBD. We did not find differences in energy intake between the groups. Our results are opposite to those of the study conducted by Weng et al. They reported that patients suffering from IBD had a lower intake of folic acid than the healthy control. However, similar to our study, they did not find differences in energy intake between groups [25]. Nevertheless, in our study, patients with IBD presented lower BMIs and body masses than the healthy group, but chronic inflammation could increase energy demand. According to European Society of Clinical Nutrition and Metabolism (ESPEN) guidelines, the energy required for patients with IBD is similar to that of a healthy population. Therefore, we could assume the energy demand is similar among the groups. However, the IBD and healthy control groups were not homogenous in BMI, so the energy demand could be different between the groups. The lack of difference in calorie intake indicates that the percentage of participants meeting the energy demand could differ between the groups.

We did not find differences in the folate intake between the CD, UC, and control groups. It is vital to notice that the lack of differences between the groups in folic acid is probably not a result of a high consumption of folate by patients suffering from IBD, but of low intake of folate by healthy controls, especially among women. Only 57% of the women with CD, 67% of the women with UC, and 64% of the healthy women met the RDA. Among men, the highest frequency of meeting the RDA occurred in the control group (88%). This was probably the consequence of a low intake of green leafy vegetables, eggs, nuts, and other vegetables or legumes, which was confirmed in Lambert et al.’s meta-analysis, where patients with IBD were found to have too low an intake of fruits, vegetables, and cereals [13].

A proper folate intake may protect against many diseases in both healthy individuals and those with IBD. Firstly, in women, it prevents neural tube defects in offspring [26]. Additionally, women with IBD planning pregnancy and treated with sulfasalazine should intake 2 mg-higher doses of folic acid [27]. Secondly, an insufficient intake of folate leads to megaloblastic anaemia [28]. On the other hand, we assessed folate intake without evaluating serum folic acid concentrations in this study. Possibly, despite a similar intake of folic acid, patients with IBD present lower folic acid levels due to higher demand [29]. Additionally, a low intake of folate may increase the homocysteine level [30], which may be unfavourable for patients with IBD since an in vitro study showed that homocysteine increased the differentiation of CD4+ into Th17 cells [31].

Our study showed that the patients with CD and UC presented lower BMDs, T-scores, and Z-scores of the FN and L1–L4 than the healthy patients. Similar results were also presented in our previous studies [18,32,33]. Osteopenia and osteoporosis are common among patients with IBD and affect about 20–50% of women and men with CD and UC. However, screening for low BMD is recommended only for patients in the risk group, i.e., treated with steroids over three months, low-trauma fracture, old age, or postmenopausal state [34]. Osteopenia and osteoporosis are already diagnosed among newly diagnosed patients [35]. Additionally, patients with IBD have a higher risk of fractures in the spine [36]. Low BMD in IBD is multifactorial. The most discussed factors in osteoporosis development are steroid therapy and chronic inflammation. However, malnutrition, malabsorption, or insufficient intake may also affect bone health [18]. An adequate calcium intake, smoking cessation, and weight exercises are recommended for osteoporosis prevention. Additionally, patients with IBD treated with steroids or with a T-score below −1.5 should be supplemented with vitamin D and calcium [34].

The RANKL/RANK/OPG (Receptor Activator for Nuclear Factor κ B Ligand/Receptor Activator of Nuclear Factor κ B/Osteoprotegerin) pathway plays an important role in bone turnover, and it is activated by excessive inflammation in IBD [37]. Vitamin B, including vitamin B5 and folic acid, also play a role in osteoclastogenesis by influencing the RANKL/RANK/OPG pathway. The study showed that both high and low concentrations of vitamin B5 affect RANKL, including osteoclast differentiation [38]. On the other hand, folic acid may prevent pregnancy-associated osteoporosis. Maroufi et al. showed that high doses of folic acid in pregnancy increase OPG levels and decrease RANKL and RANKL/OPG ratios [39].

Interestingly, we did not find differences in covering folate’s RDA among the patients who were supplemented with folic acid and those who were not. We supposed that patients supplemented with folic acid meet the folate demand through their usual diet less often than patients without supplementation. On the other hand, maybe folic acid supplementation was not associated with folic acid deficiency but with the strategy of treating or preventing anaemia and protecting against colorectal cancer. The study showed that folic acid supplementation protects against colorectal cancer [16]. Therefore, clinicians might introduce folic acid supplementation for chemoprevention, independent of nutritional status [16]. However, the European Crohn’s and Colitis Organisation (ECCO) did not recommend routine folic acid supplementation [40]. However, they suggest assessing folic acid levels at least once a year if macrocytosis is present or if the patient is being treated with thiopurine [41]. According to the ESPEN guidelines, only selected patients with IBD need folic acid supplementation [15].

On the other hand, folic acid supplementation is recommended for all Polish women of reproductive age by the Polish Society of Gynecologists and Obstetricians. Moreover, doses of folic acid for women with malabsorption, including women with CD and UC, should be twice as high as for healthy women and amount to 0.8 mg per day [42]. Therefore, our study shows that not all women met this recommendation.

Moreover, there were no significant differences in the BMDs, T-scores, and Z-scores of the FN and L1–L4 depending on the folate demand or meeting the RDA of folic acid supplementation. The lower BMD in Crohn’s disease and ulcerative colitis is multifactorial [43], and folic acid supplementation and folate intake are not the only factors affecting bone tissue. Additionally, the folic acid status may be affected by other factors—not only diet—such as the gut microbiota [44] or inflammation, which also may affect nutritional status [45]. Clements et al. reported that low-dose vitamin B (folate, vitamins B2, B6, and B12) supplementation for two years did not affect the BMD in adults over 50 years old [46].

Moreover, diet may modulate the gut microbiota, which affects the IBD course and bone tissue [47]. Alteration in the gut microbiosis is associated with IBD. The bacteria associated with IBD are *Escherichia coli*, *Bacteroides fragilis*, *Ruminococcus gnavus*, *Faecalibacterium prausnitzii*, and *Roseburia* [48]. Additionally, some bacteria in the gut microbiome may produce vitamins, including vitamin B9. Well-known bacteria-genera-synthetizing vitamins B are *Bacteroides*, *Bifidobacterium*, and *Enterococcus* [44]. Diets with a high intake of inulin-rich vegetables increase in bificobateria [49]. Therefore, a diet rich in vegetables may increase the supply of folate and modify the gut microbiota, increasing the amount of genera-producing vitamins.

The active form of folic acid is 5-methylenetetrahydrofolate, a product of folate metabolism that depends on MTHFR. Some variants of the *MTHFR* gene decrease MTHFR activity [50]. Our previous study did not find significant differences in the frequencies of the MTHFR 677 and 1298 variants in the CD, UC, and control groups. Moreover, the folic acid concentration was not different among participants with different variants of MTHFR 677 and 1298 [51].

Moreover, folic acid intake may affect BMD. In the CD group, we found a positive correlation between the folate intake per 1000 kcal and the BMD and T-score of L1–L4. Zheng et al. also found a positive correlation between folate intake and bone mineral density, especially among the population over 80 years old [52]. It is also vital to note that the frequency of vegetable consumption was positively associated with the T-score of the FN and L1–L4 [53]. Moreover, the study showed that women with osteoporosis had lower folic acid concentrations than women with normal BMDs [54]. According to Bozkurt et al., folic acid levels were not associated with osteoporosis [55].

It is vital to notice that products especially rich in folate (vegetables and fruits) are also good sources of phytochemicals, which may also affect bone tissue. Marini et al. studied the impact of genistein on the BMD in postmenopausal women with osteopenia. Their results confirmed the positive impact of a twenty-four-month treatment with genistein on the BMD [56]. On the other hand, genistein supplementation may also be useful among women with osteoporosis [57]. Moreover, isoflavones may be beneficial for bone as well as the course of disease by modulating the gut microbiota and affecting the intestinal barrier or inflammation signal pathways [58]. The chemical compounds of soy beans seem to be especially beneficial [59].

Despite the lack of significant differences, we should not exclude the impact of folate intake and folic acid supplementation on bone tissue in IBD. We found a positive correlation between folate intake and BMI and the intake of folate per 1000 kcal and the BMD, T-score, and Z-score of L1–L4. A better nutritional status is probably associated with both a higher folate intake and BMI [60]. On the other hand, a higher intake of folate per 1000 kcal may be due to a higher consumption of vegetables and fruit associated with a better disease course. In turn, a better course of disease is caused by less inflammation, which affects bones positively. However, Stone et al. showed that vitamin B12, B6, and folic acid supplementation did not affect the risk of fracture or bone metabolism among women with hyperhomocysteinemia or deficiencies of vitamins B12, B6 or folate [61].

**Figure 1:**
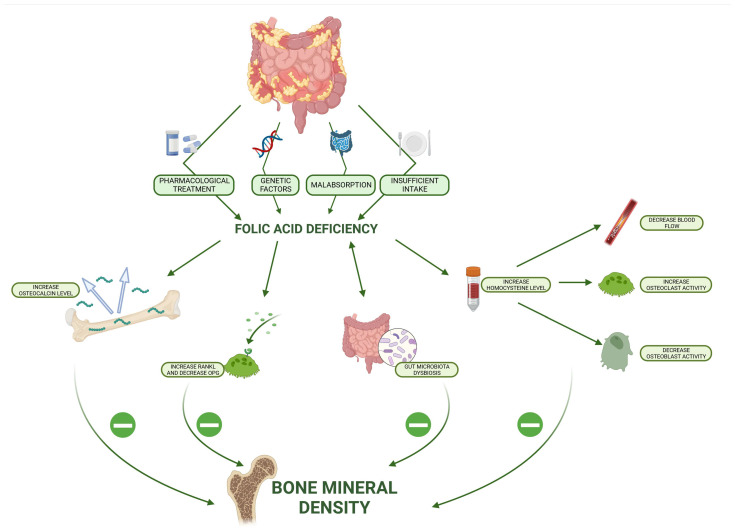
Possible mechanisms for impact of folic acid deficiency on bone mineral density in inflammatory bowel disease (RANKL—Receptor Activator for Nuclear Factor κ B Ligand; OPG—osteoprotegerin).

All possible mechanisms for the impact of a folic acid deficiency are presented in Figure 1.

Although we paid attention to choosing proper methods in our study, some limitations can be found. Firstly, the number of patients in the study is not significant. However, collecting data based on a 24 h dietary recall is not straightforward; patients refuse to complete this questionnaire. Secondly, we only assessed the intake of folate without measuring folic acid levels. Next, we did not consider pharmacological treatment or the course of disease, which may affect folic acid demand. Additionally, we did not specify the season of our study. The availability of fresh vegetables and fruit rich in folate is different in spring, summer, fall, and winter. We did not include information about physical activity. However, our previously study showed a lack of differences in physical activity between groups and no association between physical activity and BMD [62].

## 5. Conclusions

In conclusion, the patients with IBD have a higher risk of osteoporosis development when compared with the control group. Therefore, we must look for new risk factors and preventive actions for osteoporosis development. Moreover, there are no significant differences in the BMDs, T-scores, and Z-scores of the FN and L1–L4 in patients with IBD depending on them meeting the folate demand. However, the positive correlation between folate intake and BMD indicates the potential impact of folate on bone health. We need further studies on the association between folate intake and supplementation, serum levels of folic acid, and the BMD. Nevertheless, patients should be encouraged to consume products rich in folate according to their personal tolerance. Our study shows that many patients with CD and UC and healthy adults consume too little folate.

We need to look for new strategies for osteoporosis prevention in IBD. Based on our study and the research of other authors [63], a well-balanced diet, including the intake of all nutrients and phytochemicals as well as proper physical activity, seems to be an important element for the protection of bone mass.

## Figures and Tables

**Table 1:** Characteristics of the study groups.

Parameter	CD (n = 26)	UC (n = 30)	CG (n = 31)	*p*-Value
Age [years]	30.50 (25.40; 37.20)	28.20 (21.90; 37.60)	32.90 (27.10; 42.90)	0.11
Body mass [kg]	59.70 (50.80; 65.90)	58.60 (53.30; 69.30)	67.60 (61.50; 78.80)	<0.01 <0.01 ^a^ 0.02 ^b^ 0.99 ^c^
BMI [kg/m^2^]	19.95 (19.10; 23.10)	19.90 (17.70; 22.40)	23.00 (20.90; 25.90)	<0.01 0.04 ^a^ <0.01 ^b^ 0.99 ^c^
BMD (L1–L4) [g/cm^2^]	1.136 (1.077; 1.235)	1.160 (0.984; 1.259)	1.280 (1.172; 1.367)	<0.001 <0.01 ^a^ <0.01 ^b^ 0.99 ^c^
T-score (L1–L4)	−0.400 (−1.000; 0.500)	−0.200 (−1.700; 0.700)	0.700 (−0.100; 1.300)	<0.001 <0.01 ^a^ <0.01 ^b^ 0.99 ^c^
Z-score (L1–L4)	−0.300 (−0.600; 0.600)	−0.400 (−1.300; 0.900)	0.400 (0.100; 1.400)	<0.001 <0.01 ^a^ <0.01 ^b^ 0.99 ^c^
BMD (femoral neck) [g/cm^2^]	0.987 (0.938; 1.135)	1.038 (0.837; 1.147)	1.097 (1.048; 1.192)	<0.01 0.02 ^a^ 0.02 ^b^ 0.99 ^c^
T-score (femoral neck)	−0.450 (−0.800; 0.600)	0.050 (−1.200; 1.00)	0.400 (0.100; 1.100)	<0.01 0.03 ^a^ 0.02 ^b^ 0.99 ^c^
Z-score (femoral neck)	0.050 (−0.400; 0.600)	0.050 (−1.200; 1.000)	0.600 (0.200; 1.200)	<0.01 0.04 ^a^ 0.02 ^b^ 0.99 ^c^
Folate intake [mg]	226.77 (191.27; 352.86)	273.50 (205.42; 415.75)	302.63 (226.93; 393.92)	0.23
Meet the RDA [%]	56.69 (47.82; 88.21)	68.38 (51.35; 103.95)	75.66 (56.73; 98.48)	0.23
Energy intake [kcal]	1720.415 (1401.15; 2151.87)	1837.10 (1423.77; 2287.49)	1738.74 (1504.08; 2274.53)	0.58
Folate/1000 kcal [mg/kcal]	145.89 (123.04; 163.93)	171.05 (120.67; 185.68)	155.058 (131.98; 181.57)	0.43
CRP	5.85 (1.40; 21.80)	2.70 (1.10; 10.30)	0.70 (0.30; 1.70)	<0.0001 <0.0001 ^a^ <0.001 ^b^ 0.97 ^c^

CD—Crohn’s disease; UC—ulcerative colitis; CG—control group; BMI—body mass index; BMD—bone mineral density; RDA—recommended daily allowance; CRP—C-reactive protein; ^a^—CD vs. CG; ^b^—UC vs. CG; ^c^—CD vs. UC.

**Table 2:** Comparing intake of energy and folate per 1000 kcal and meeting the RDA for folate in patients with Crohn’s disease, patients with ulcerative colitis, and the control group.

Sex	Parameter	CD	UC	CG	*p*-Value
Women	Number of patients [n]	16	16	19	
	Meet the RDA [%]	56.69 (48.68; 84.26)	67.25 (52.68; 86.86)	63.50 (53.45; 90.41)	0.78
	Energy intake [kcal]	1552.23 (1320.17; 1770.53)	1551.89 (1378.58; 1772.36)	1601 (1421.58; 1721.16)	0.73
	Folate/1000 kcal [mg/kcal]	153.66 (139.16; 196.74)	184.52 (157.82; 192.18)	155.06 (131.98; 189.44)	0.46
Men	Number of patients [n]	10	14	12	
	Meet the RDA [%]	62.21 (47.28; 88.56)	78.06 (51.35; 104.02)	88.23 (63.82; 102.56)	0.21
	Energy intake [kcal]	2120.23 (1866.95; 2272.05)	2304.79 (2003.21; 2423.36)	2229.51 (1946.44; 2573.28)	0.57
	Folate/1000 kcal [mg/kcal]	119.96 (106.91; 139.86)	139.81 (114.51; 183.29)	149.34 (130.89; 175.36)	0.17

CD—Crohn’s disease; UC—ulcerative colitis; CG—control group; RDA—recommended daily allowance.

**Table 3:** Frequency of folic acid supplementation in women and men with normal and insufficient folate intake.

Sex		Inadequate Intake (below 100% of RDA)	Adequate Intake (at least 100% of RDA)	*p*-Value
Women				
Supplementation	yes	12 (85.71%)	2 (14.29%)	0.70
	no	30 (81.08%)	7 (18.92%)	
Men				
Supplementation	yes	7 (63.64%)	4 (36.36%)	0.30
	no	20 (80.00%)	5 (20.00%)	

RDA—recommended daily allowance.

**Table 4:** Comparing specific parameters depending on meeting the folate demand in patients with Crohn’s disease and ulcerative colitis, and control group.

	CD			UC			CG		
	Insufficient (n = 24)	Normal (n = 2)	*p*-Value	Insufficient (n = 21)	Normal (n = 9)	*p*-Value	Insufficient (n = 24)	Normal (n = 7)	*p*-Value
BMI [kg/m^2^]	19.85 (19.00; 22.15)	23.90 (23.10; 24.70)	0.16	19.60 (17.70; 21.50)	20.40 (19.70; 24.90)	0.15	22.60 (20.85; 25.50)	23.60 (22.00; 24.70)	0.63
BMD (L1–L4) [g/cm^2^]	1.136 (1.047; 1.218)	1.243 (1.095; 0.391)	0.56	1.154 (0.989; 1.267)	1.165 (0.984; 1.246)	0.98	1.276 (1.172; 1.335)	1.335 (1.226; 1.545)	0.18
T-score (L1–L4)	−0.400 (−1.200; 0.350)	0.400 (−1.000; 1.800)	0.60	−0.200 (−1.700; 0.700)	−0.100 (−1.900; 0.200)	0.98	0.550 (−0.200; 1.250)	1.300 (0.100; 3.000)	0.15
Z-score (L1–L4)	−0.300 (−0.550; 0.400)	0.500 (−0.900; 1.900)	0.74	−0.400 (−1.300; 0.900)	−0.400 (−1.300; 0.200)	0.87	0.400 (−0.050; 1.150)	0.800 (0.200; 3.200)	0.20
BMD (femoral neck) [g/cm^2^]	0.987 (0.934; 1.129)	1.158 (0.963; 1.353)	0.36	1.032 (0.837; 1.148)	1.063 (0.868; 1.109)	0.96	1.095 (1.036; 1.174)	1.102 (1.093; 1.255)	0.21
T-score (femoral neck)	−0.450 (−0.900; 0.550)	0.750 (−0.800; 2.300)	0.56	−0.300 (−1.700; 0.800)	0.200 (−1.600; 0.300)	0.91	0.300 (0.000; 0.850)	0.500 (0.200; 1.600)	0.19
Z-score (femoral neck)	0.050 (−0.350; 0.550)	0.850 (−1.000; 2.700)	0.89	0.100 (−1.200; 1.200)	0.000 (−1.200; 0.200)	0.72	0.450 (0.200; 1.100)	0.800 (0.400; 2.300)	0.21
CRP	5.85 (1.45; 19.95)	11.10 (0.40; 21.80)	0.67	3.10 (1.10; 10.80)	2.30 (1.80; 7.30)	0.87	0.80 (0.40; 1.70)	0.30 (0.20; 0.50)	0.10

CD—Crohn’s disease; UC—ulcerative colitis; CG—control group; BMI—body mass index; BMD—bone mineral density, CRP—C-reactive protein.

**Table 5:** Comparing specific parameters depending on folic acid supplementation in patients with Crohn’s disease, ulcerative colitis and the control group.

	Folic Acid Supplementation					
	CD			UC		
	Yes (n = 11)	No (n = 15)	*p*-Value	Yes (n = 13)	No (n = 17)	*p*-Value
BMI [kg/m^2^]	22.10 (18.80; 23.40)	19.40 (19.10; 20.80)	0.30	19.70 (18.60; 21.30)	20.20 (17.70; 22.40)	0.88
BMD (L1–L4) [g/cm^2^]	1.136 (1.096; 1.287)	1.126 (1.011; 1.235)	0.31	1.154 (1.072; 1.259)	1.165 (0.972; 1.246)	0.52
T-score (L1–L4)	−0.400 (−1.000; 0.700)	−0.500 (−1.700; 0.500)	0.41	−0.200 (−1.200; 0.700)	−0.200 (−1.800; 0.500)	0.62
Z-score (L1–L4)	−0.100 (−0.600; 0.600)	−0.400 (−1.200; 0.600)	0.36	−0.200 (−0.500; 0.900)	−0.500 (−1.300; 0.500)	0.33
BMD (neck) [g/cm^2^]	0.998 (0.942; 1.145)	0.980 (0.903; 1.074)	0.41	1.043 (0.857; 1.131)	0.968 (0.837; 1.232)	0.93
T-score (neck)	−0.500 (−0.700; 0.800)	−0.400 (−1.00; 0.300)	0.48	0.000 (−1.600; 0.600)	−0.500 (−1.800; 1.400)	0.97
Z-score (neck)	0.200 (−0.100; 0.800)	0.000 (−0.700; 0.200)	0.45	0.100 (−1.200; 0.900)	−0.400 (−1.400; 1.500)	0.98
CRP	5.40 (1.30; 11.60)	6.30 (2.30; 26.80)	0.41	1.30 (0.60; 7.30)	6.00 (2.00; 10.30)	0.19

CD—Crohn’s disease; UC—ulcerative colitis; BMI—body mass index; BMD—bone mineral density; CRP—C-reactive protein.

## Data Availability

Data and materials are available upon contact with the corresponding author.
